# Deep Learning-Based Detection of Honey Storage Areas in *Apis mellifera* Colonies for Predicting Physical Parameters of Honey via Linear Regression

**DOI:** 10.3390/insects16060575

**Published:** 2025-05-29

**Authors:** Watit Khokthong, Panpakorn Kritangkoon, Chainarong Sinpoo, Phuwasit Takioawong, Patcharin Phokasem, Terd Disayathanoowat

**Affiliations:** 1 Department of Biology, Faculty of Science, Chiang Mai University, Chiang Mai 50200, Thailand; watit.khokthong@cmu.ac.th (W.K.); chainarong.s@cmu.ac.th (C.S.); patcharin.ph@cmu.ac.th (P.P.); 2 Research Center of Deep Technology in Beekeeping and Bee Products for Sustainable Development Goals (SMART BEE SDGs), Chiang Mai University, Chiang Mai 50200, Thailand; songkranoppa@gmail.com; 3 Bachelor of Environmental Science Program, Faculty of Science, Chiang Mai University, Chiang Mai 50200, Thailand; beckhaman@outlook.com; 4 Office of Research Administration, Chiang Mai University, Chiang Mai 50200, Thailand

**Keywords:** honeybee hive classification, deep learning, image processing, object detection, YOLO, honey quantification, automated monitoring

## Abstract

Traditional methods for assessing honey storage in beehives predominantly rely on manual visual inspection, which often leads to inconsistencies and inefficiencies. This study presents an automated deep learning approach utilizing the YOLOv11 model to detect, classify, and quantify honey cells within *Apis mellifera* frames across monthly sampling periods. The model’s performance varied depending on image resolution and dataset partitioning. Using the free version of YOLOv11 with high-resolution images (960 × 960 resolution) and a dataset split of 90:5:5 for training, validating, and testing, the model achieved a mean average precision at IoU threshold of 0.5 (mAP@0.5) of 83.4% for uncapped honey cells and 80.5% for capped honey cells. A strong correlation (r = 0.94) was observed between the 90:5:5 and 80:10:10 dataset splits, indicating that increasing the volume of training data enhances classification accuracy. In parallel, the study investigated the relationship between the physical properties of honey and image-based honey storage detection. Of the four tested properties, electrical conductivity (R^2^ = 0.19) and color (R^2^ = 0.21) showed weak predictive power for honey storage area estimation, with even weaker associations found for pH and moisture content. The honey storage areas via 90:5:5 and 80:10:10 datasets moderately correlated (r = 0.44–0.46) with increasing electrical conductivity and color. Especially, electrical conductivity exhibited statistically significant correlations with dataset performance across different dataset splits (*p* < 0.05), suggesting some potential influence of chemical composition on model accuracy. Our findings demonstrate the viability of image-based honey classification as a reliable technique for monitoring beehive productivity. Additionally, the research on image-based honey detection can be a non-invasive solution for improved honey production, beehive productivity, and optimized beekeeping practices.

## Simple Summary

This study addresses the challenge of accurately and efficiently monitoring honey production in beehives. Using high-resolution photographs captured under controlled lighting with a commercial digital camera, we analyzed honeycomb images with a deep learning model to automatically detect and quantify honey-filled cells as a proportion of the total comb area. The deep learning system performed best in identifying uncapped honey cells. Additionally, we validated the pixel-based classification results against measured honey physical properties—including pH, conductivity, moisture content, and color. Although the automated method revealed only weak correlations with these physical parameters, the deep learning-based classification offers a promising solution for real-time, scalable monitoring of hive productivity, supporting modern and data-driven beekeeping practices.

## 1. Introduction

Beekeeping has been an important part of world agriculture and environmental stewardship for centuries, with honeybees delivering valuable pollination services that enhance food production and biological diversity [1]. Western honeybees (*Apis mellifera*) play a vital role in maintaining agricultural ecosystems through pollination of flowering plants, contributing significantly to global food production from crops used for human consumption [2,3]. Given the global expansion of the beekeeping industry, a critical concern for stakeholders is the implementation of routine physical inspections in accordance with modern apicultural practices, particularly to ensure proper honey storage, maintain hive health, and enhance the quality and marketability of bee products [4]. Monitoring the health of honeybee colonies is crucial for sustainable beekeeping practices. Although physical honeybee colony inspections during the summer are very important to encourage honey production regarding time-based management, this practice should not be allowed, as it causes subsequent disturbances during the major nectar flows [4].

Traditional beehive inspections on honey storage by beekeepers often rely on visual estimation, which can be fast but inaccurate. The estimates of honey or nectar provision per comb frame are obtained by subtracting the weight of the foundation and other components (such as capped brood, larvae, and bee bread) from the total weight of the frame without bees [5,6]. To enable new functionalities in precision beekeeping—such as honey production monitoring, pollination optimization, and bee health assessment—developing intelligent hives equipped with sensors for audio and image data analysis is considered a best management practice [5,6,7,8,9]. As the cost of camera sensor systems decreases and their capabilities improve, image acquisition in beehives still presents challenges. For example, capturing high-quality images often requires the use of a wooden tunnel to block external light [10], and inspections remain time-consuming and are not typically conducted continuously. In addition, technological developments should incorporate analytical overlays to provide beekeepers with precisely processed data. Although digital images of honey and nectar cells can be successfully quantified using DeepBee^©^ software, (https://github.com/AvsThiago/DeepBee-source, accessed on 12 January 2025) the actual weight of honey or nectar varies depending on the depth of the cells [10]. Consequently, modern honeybee colony monitoring systems are expected to become more prescriptive, providing data that is analyzed before being displayed to users. This will allow beekeepers to process digital data more precisely, particularly when analyzing information from multiple colonies to support informed decision-making.

In modern apiculture, as opposed to wild nests, beehives consist of several honeycomb frames, where bees store brood, pollen, and honey [7]. Recent studies have addressed the pollen detection in beehives as well as its color variation and textures using image-based classification to enable suitable interventions and management strategies [7,11]. Counting the comb cells with bee food reserves offers information on colony health status [10]. To improve colony health assessments, methods such as CombCount, (https://github.com/jakebruce/CombCount, accessed on 12 January 2025) a semi-automated brood counting tool, enhance accuracy by efficiently detecting empty comb cells and providing more reliable estimates of brood area [12]. Furthermore, quantitative assessment of honey yield is equally important, as it reflects the productivity of the hive and directly impacts economic returns. Deep learning algorithms embedded in computer vision, which offer precise automated honey detection, are considered the next step toward improving the beekeeping industry by enabling researchers to analyze high-resolution photographs of honeycomb structures through digital image processing [7,13]. Automated segmentation techniques can estimate the proportion of comb cells filled with honey, offering a rapid and objective alternative to manual counting. Precision apicultures are relying on image databases and segmentation algorithms; however, deriving spectral signatures from low-resolution images directly still gives limited information [13,14].

In precision beekeeping, images are commonly used to train convolutional neural networks (CNNs), a technique widely adopted in deep learning algorithms for object detection [15,16]. Object detection methods are generally categorized into one-stage and two-stage approaches [17]. Redmon et al. (2016) [18] introduced YOLO (You Only Look Once), a single-stage deep learning detection algorithm. The latest version, YOLOv11, incorporates the C3K2 module to improve the accuracy of small object detection [19]. Previous CNN-based object detection models, such as ResNet and AlexNet [20], as well as region proposal-based models like Faster R-CNN [21] and Mask R-CNN [22], have been applied to computer vision-based honeybee inspections. While these two-stage models rely on region proposals and deliver strong accuracy, YOLO’s single-stage architecture enables it to perform object classification and localization within a single network, directly extracting features to make predictions [17]. Additionally, the single-shot multibox detector (SSD), another single-stage CNN algorithm, has been used in honeybee inspection systems. However, SSD has shown inferior performance compared to YOLOv5 in detecting *Varroa destructor* mites using image datasets [23]. Therefore, deep learning algorithms embedded in computer vision, offering precise automated honey detections, are considered the next step toward improved beekeeping industries.

Data augmentation is essential for enhancing the robustness of deep learning in computer vision models [14,24], such as for honey and pollen cell detection. Especially, detecting uncapped and capped brood, as well as honey cells, remains a challenging task for automated computer vision algorithms. Earlier, the circular Hough transform (CHT) demonstrated some capability in detecting honeybee cells [25,26]; however, bee comb cells are naturally hexagonal in shape, not circular. To address this limitation, object-based detection using the CHT method was enhanced with semantic segmentation techniques, as implemented in the free software DeepBee^©^ (https://github.com/AvsThiago/DeepBee-source, accessed on 12 January 2025, which can detect hexagon-shaped cells in bee combs [10]. The detection methods used in Alves et al. (2020) [10] demonstrated high accuracy in identifying honey cells by distinguishing between cells containing eggs, larvae, capped brood, pollen, nectar, honey, and other materials. To improve feature extraction for hexagonally shaped honey cells, data augmentation plays a crucial role in enhancing deep learning models in computer vision [24]. The X-AnyLabeling v.2.5.3 tools, built on the PyPI package, support a wide range of annotation shapes, such as freeform multi-vertex polygons, which facilitate detailed data annotation and more accurate feature extraction of the YOLO base models.

Honey quality is influenced by various physical and chemical properties, including pH, electrical conductivity, moisture content, and color. These parameters are often used to assess honey’s freshness, stability, and purity, which depend primarily on the botanical origin—determined through pollen analysis [27]—as well as on microbiological properties [28,29]. While these factors and conditions play a crucial role in the honey properties, the extent to which the honey area—extracted from image segmentation—affects these properties remains unclear. Our aims were to (1) apply digital image processing and deep learning to enhance precision beekeeping by determining the percentage of honey area within the beehive, and (2) explore its relationship with physical parameters. The four measured variables are pH, electrical conductivity (EC), moisture content, and color, which could investigate the feasibility of applying the honey data via deep learning in honeybee products and yield estimation. This information will help beekeepers and researchers assess comb conditions in honeybee farms in Thailand, with potential applications in other countries as well.

## 2. Materials and Methods

### 2.1. Dataset

#### 2.1.1. Experimental Setup

**Figure 1:**
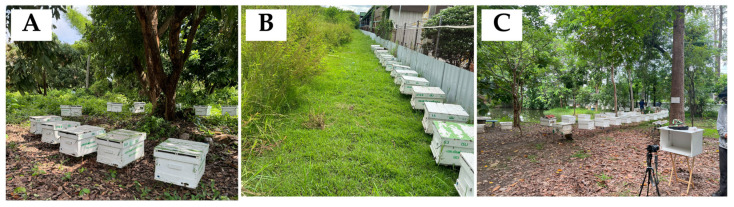
Apiary locations (**A**) Agricultural Technology Promotion Center for Economic Insects, (**B**) Chiang Mai Healthy Product Co., Ltd. (Chiang Mai, Thailand), and (**C**) Faculty of Agriculture, Chiang Mai University.

The experiment was conducted over six months (July 2024–January 2025, except October 2024), in Chiang Mai province, Thailand. The setup for this research involved visits to three apiary locations (Figure 1): Agricultural Technology Promotion Center for Economic Insects (coordinates: 18.73729, 98.92272), Chiang Mai Healthy Product Co., Ltd., 193 (coordinates: 18.68635, 99.05318), and Faculty of Agriculture, Chiang Mai University (coordinates: 18.79348, 98.96000). At each site, data were collected from queenright *Apis mellifera* colonies, each consisting of 8 to 10 frames per colony. All locations were selected for their active beekeeping practices and diverse colony compositions, which provided ideal conditions for controlled data collection of every single frame, ensuring a robust dataset for analysis.

#### 2.1.2. Image Acquisition

**Figure 2:**
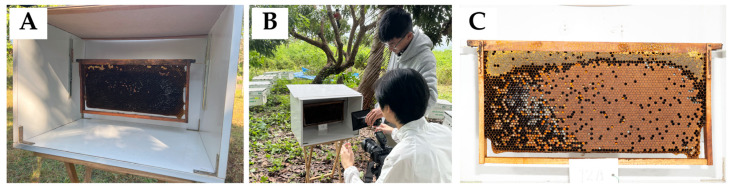
Experimental setups: (**A**) the *Apis mellifera* frame is placed on the holders inside the studio box, (**B**) the researcher made adjustments to the camera before image capture, and (**C**) an example of a frame from the image captured.

To ensure consistent lighting conditions, a portable DIY wooden studio box (Figure 2) was used along with a Flash Godox TT685 TTL. A digital camera, Sony A7R4, was employed for image capture. The camera was placed 50 cm from the beehive, and image samples were collected from the two-sided beehives. During the process, the studio box was closed on the other sides to minimize external light interference. The images captured had a resolution of 60 megapixels (9504 × 6336 pixels) without adult bees on the frame. In this study, four *A. mellifera* colonies were selected from each site. From each colony, four frames were chosen, resulting in a total of 16 frames per site. All selected frames were photographed on both sides, yielding 96 images per visit across the three sites. Data collection was conducted monthly using the same frames, resulting in a total of 464 high-resolution images over the entire study. However, not all captured images were used for this study, as some frames lacked honey and due to the flood event in Chiang Mai in October 2024 (Upper Northern Region Irrigation Hydrology Center, https://www.hydro-1.net, accessed on 12 January 2025). The final dataset that was analyzed consisted of 300 images that met the necessary criteria for this research.

#### 2.1.3. Image Annotation

**Figure 3:**
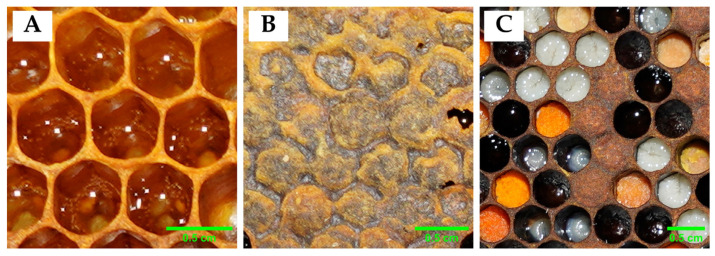
Comb by differentiating classes (**A**) uncapped honey cells, (**B**) capped honey cells, and (**C**) others (empty cell, pollen, larva, and pupa).

A total of 300 images were annotated by using X-AnyLabelling v.2.5.3 and uploaded into the Roboflow platform. The annotation of the images involved drawing a polygon around the objects of interest and assigning object classes (Figure 3) to the polygon. Table 1 shows all of the different classes annotated in the dataset.

To calculate the percentage of honey in a honeycomb, it is essential to capture both the honey class and the total area of the honeycomb. While the honey class alone is sufficient to identify the honey-filled cells, the total area of the honeycomb is needed to determine the proportion of honey relative to the entire structure. To do this, we differentiate between visible and uncapped types of honey cells as one of the three formal types of cells, which was tested with machine learning algorithms [30].

**Figure 4:**
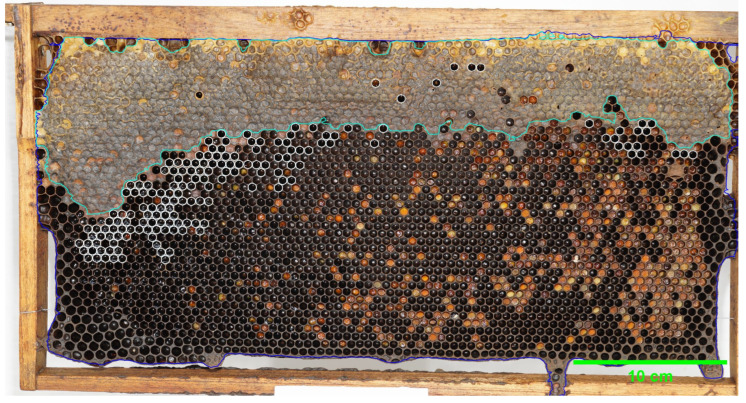
Data annotation consists of uncap (cyan polygon), cap (white polygon), total (blue polygon) from the frame sample ID: _DSC5335 (20 December 2024).

The uncapped class has the highest number of instances because the selected images exclusively feature honeycombs. Given that honeycombs consist of numerous cells, the number of honey-filled cells is naturally high. After annotating and labeling all images, they were exported to the YOLO format with the configuration [class_id x, y, w, h]. These parameters are used to represent an object in a computer vision system. The annotation files were saved with the same names as the images, and the configurations for the file paths that feed the model for training, validating, and testing were then saved in a YAML file. Figure 4 shows an example of an annotation image on Roboflow.

#### 2.1.4. Data Augmentation

Before model training, the annotated dataset was preprocessed and resized to five distinct dimensions: 960 × 960, 800 × 800, 640 × 640, 512 × 512, and 256 × 256 resolution. This resizing ensured compatibility with the input requirements of the pre-trained models. Additionally, data augmentation techniques were employed to increase the dataset size and improve generalization by reducing the risk of overfitting. These techniques create new training examples for your model to learn from by generating augmented versions of each image in your training set. Such as flipping, rotating, and altering image properties, a process that effectively enhances dataset diversity. Table 2 summarizes the preprocessing and augmentation settings applied.

### 2.2. Object Detection Model

#### Model Training and Validation

This study utilized YOLOv11s-seg frameworks for object detection and instance segmentation. The dataset was divided into training portions (90%, 80%, 70%), validating portions (5%, 10%, 15%), and testing portions (5%, 10%, 15%) with three subsets; 90:5:5, 80:10:10 and 70:15:15. YOLOv11, the latest version of the YOLO algorithm, released by Ultralytics on 30 September 2024 was used.

The training was conducted on an NVIDIA GeForce 3070 laptop GPU with 8 GB of GDDR6 memory, NVIDIA Ampere with 5120 CUDA Cores, and 40 RT Cores with significantly low TOPS (~20–25 TFLOPS FP32) but still capable of AI tasks. Pretrained weights from the COCO dataset were leveraged to accelerate and improve the training process. The model was trained using various combinations of input image sizes and batch sizes and tested across multiple YOLOv11 architectures as previously described. The input image sizes included 960 × 960, 640 × 640, 512 × 512, and 256 × 256 pixels, while batch sizes of 4. The performance of these combinations was evaluated to determine the optimal model and parameter settings for real-time applications. To eliminate this source of variance and enable direct performance comparison, we fixed all training runs to exactly 200 epochs, regardless of individual early-stop points.

### 2.3. Data Extraction

**Figure 5:**
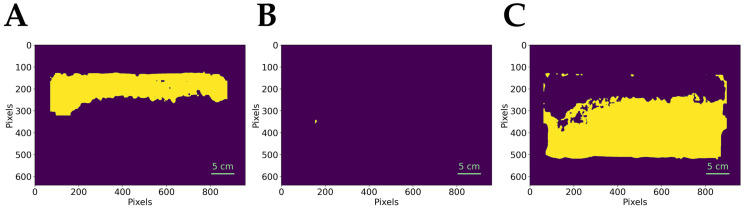
Extracted data from sample ID: _DSC5335 (20 December 2024) (**A**) capped honey cells, (**B**) uncapped honey cells, and (**C**) others. **Yellow represents the extracted regions of interest, and purple indicates the background**.

After predicting the image, we extract the segmented area of each class from the predicted image and measure its region properties using the scikit-image. This process enables the calculation of the honey percentage by analyzing the honey area in pixels. Figure 5 shows an example of an extracted data plot by matplotlib.

### 2.4. Performance Metrics

After completing the training and validation phases, the performance of the models is assessed by testing them on a designated test dataset. Selecting appropriate evaluation metrics for object detection models can be complex.

#### 2.4.1. Precision and Recall

Precision measures the proportion of correctly predicted instances, reflecting the model’s reliability in producing accurate predictions. Recall, on the other hand, quantifies the proportion of relevant instances correctly identified by the model. These metrics are calculated using the following equations:

*P* = *TP*/(*TP* + *FP*) (1)

*R* = *TP*/(*TP* + *FN*) (2)

Here, *TP* represents true positives (correctly detected objects), *FP* indicates false positives (incorrectly predicted objects), and *FN* denotes false negatives (missed detections). A detection is classified as a *TP* if the Intersection over Union (*IoU*) between the predicted bounding box and the ground truth exceeds a predetermined threshold (commonly 0.5). Otherwise, it is considered an *FP*.

#### 2.4.2. Average Precision and Mean Average Precision

Average precision (AP) is computed as the area under the precision-recall curve, as defined by the following:

(3) $$AP=\sum_{k=0}^{n-1}\left[Recall(k)-Recall(k+1)]\times Precision(k\right)$$

In Equation (3), *n* denotes the total number of discrete precision–recall evaluation points (one for each unique detection-score threshold), and *k* indexes each of those points from 0 to *n*–1. The *IoU*, which evaluates the overlap between the detected and actual bounding boxes, is given by the following:

(4) $$IoU=\frac{Area\;of\;Overlap\;between\;Object\;and\;Detected\;Box}{Area\;of\;Union\;between\;Object\;and\;Detected\;Box}$$

The *AP* score ranges between 0 and 1, providing a single value that summarizes precision across recall levels. Mean average precision (*mAP*) is calculated as the mean of *AP* values across all object classes:

(5) $$mAP=\frac{1}{n}\sum_{i=1}^{n}{AP}_{i}$$

In this expression, n refers to the total number of object classes under evaluation, and i runs from 1 to *n*, indexing each class’s individual average-precision score (*AP*_1_…*AP_n_*) before they are averaged to yield *mAP*. Object detection models often use two *mAP* thresholds: *mAP@0.5* (the mean *AP* for an *IoU* threshold of 0.5) and *mAP@0.5:0.95* (the mean *AP* averaged over *IoU* thresholds from 0.5 to 0.95). In this study, the performance of all selected models was evaluated using precision, recall, and *mAP@0.5*, which are widely recognized as standard benchmarks for evaluating object detection models.

#### 2.4.3. Honey Area Acquisition and Dataset Fittings Along Linear Regression Line

The percentage of honey area was computed by comparing pixel counts of honey presence to the total hive area. This method aligns with ongoing research in automated beehive monitoring by the following equation:

(6) $$Honey\;area\;\left(\%\right)=\;[(Capped+Uncapped)/Total]\times 100$$

**Figure 6:**
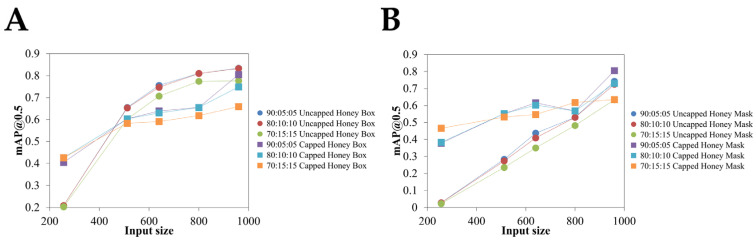
Testing accuracy according to the different image input sizes: (**A**) bounding box, and (**B**) mask.

**Figure 7:**
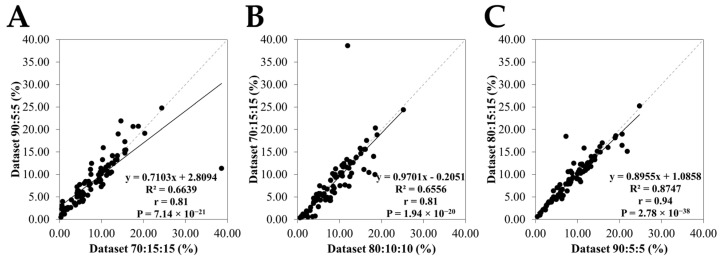
Honey areas estimated from the selected 960 × 960 resolution splits (**A**) 90:5:5 vs. 70:15:15, (**B**) 70:15:15 vs. 80:10:10, and (**C**) 80:10:10 vs. 90:15:15. The equality line (gray dashed line) indicates a 1:1 relationship.

Image datasets were processed to retrieve pixel data from different sides of the hive (Side A, Side B, and Both Sides) contribute to the calculation of honey area percentage (Supplementary Table S2). Here, we selected the 960 × 960 resolution because it gives the highest mAP@0.5 results (Table 3, Figure 6). When the model with 960 × 960 resolution was trained and tested under three different data-splitting schemes: 90:5:5, 80:15:15, and 70:15:15, the scatter plot compared the percentage of honey area estimated by one dataset split on the *x*-axis to another split on the *y*-axis. The fitted regression lines, along with their corresponding slopes, intercepts, the Pearson correlation coefficient (r), coefficient of determination (R^2^), and *p*-values, quantified how well these different splits agree with each other (Figure 7).

### 2.5. Assessment and Measurement of Honey Physical Parameters in Honeybee Hives

We took 20 mL of the honey sample from each beehive after the photography to test four physical parameters. Since the pH of honey influences its flavor profile, fermentation rate, and microbial stability, and moisture content is a key parameter influencing honey’s shelf life, viscosity, and susceptibility to fermentation [31]. Electrical conductivity provides insights into the mineral and organic acid content of honey [32] and also help identify adulteration or contamination, thus ensuring honey purity [33]. Color is one of the key indicators of honey type that is often linked to floral origin and consumer preference. This measurement supports both quality control and product marketing [34]. Here, we tested pH, EC, moisture content, and color for all honey samples, then fit them in the linear regression with estimated honey storage areas provided from data-splitting schemes: 90:5:5, 80:15:15, and 70:15:15.

#### 2.5.1. pH Measurement

The pH of honey samples was measured under controlled laboratory conditions using a digital benchtop pH meter (SUNTEX SP-2100, Taipei, Taiwan). The meter comprises a sensitive electrode probe and a digital display unit, allowing precise and rapid measurement of honey samples or hive-derived fluids. For each honey sample, four grams of honey were mixed with 30 mL of DI water until a homogeneous solution was obtained. The pH was then measured, with three replications performed for each sample.

#### 2.5.2. Electrical Conductivity (EC)

The HI99300 Hanna meter (Hanna Instruments, Nusfalau, Romania) was used to measure the EC of honey using a sample of four grams dissolved in 20 mL of DI water. These portable meters feature a probe that detects the ionic content of the solution, displaying results in millisiemens per centimeter (mS/cm) or parts per million (ppm).

#### 2.5.3. Moisture Content

A digital handheld honey refractometer (ATAGO PAL-22S, Tokyo, Japan) was employed to measure the moisture content. In this device, a small sample of honey is placed on the sensor, and an internal light source measures the refraction of the sample, which is then correlated to moisture or Brix values.

#### 2.5.4. Color Measurement

The HI96785 Honey Color Photometer (Hanna Instruments, Nusfalau, Romania) measured the honey color on the Pfund scale, which ranges from water-white to dark amber. This device uses a tungsten lamp and a silicon photodetector to determine the transmittance of the sample, thereby classifying the honey’s color.

## 3. Results

### 3.1. Model Performance

The model’s performance in detecting capped and uncapped honey cells was evaluated across different image sizes and data splits using mAP@0.5 as the primary performance metric. A dataset of 300 images was used to assess the effectiveness of the models under varying conditions. Table 3 and Figure 6 show the impact of image resolution and data split on model performance for detecting uncapped and capped honey cells, where the 960 × 960 resolution of input images depicted more mAP@0.5 values than the other resolutions.

### 3.2. Comparison of Honey Area Estimates Among Different Datasets

Figure 7 illustrates the relationships among the honey area predictions. In Figure 7A, the regression between the 90:5:5 and 70:15:15 is described by the equation y = 0.7103x + 2.8094. The correlation coefficient (r = 0.81) and the coefficient of determination (R^2^ = 0.66). In Figure 7B, the analysis comparing the 80:10:10 and 70:15:15 yielded the regression line y = 0.9701x − 0.2051 with a correlation coefficient of r = 0.81 and R^2^ was 0.66. In Figure 7C, a comparison between the 90:5:5 and 80:10:10 obtained the regression equation y = 0.8955x + 1.0858, along with a correlation coefficient of r = 0.94, indicating a strong linear relationship. The equality line (y = x) fitted well to this data-splitting scheme (R^2^ value of 0.87, *p* < 0.01), near one-to-one correspondence, which demonstrates that the 80:10:10 split yielded predictions that are highly consistent with those of the 90:5:5 split.

### 3.3. Relationship Between the Physical Parameters of Honey and the Honey Area

**Figure 8:**
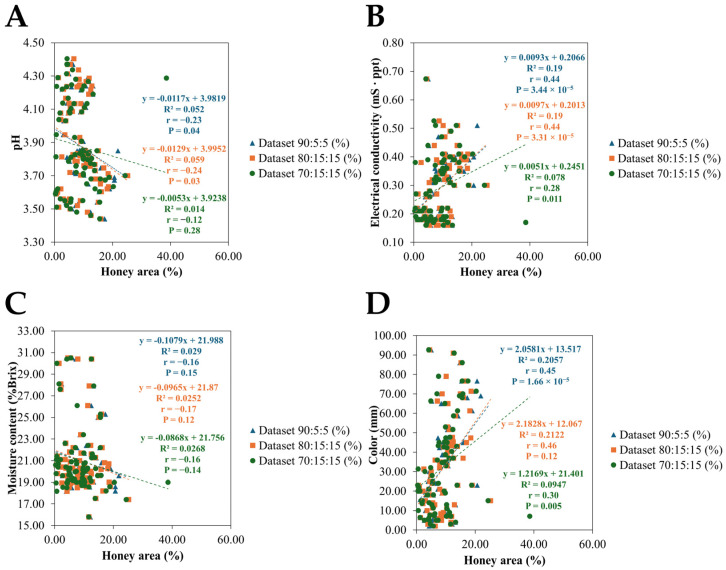
Correlation between honey area (%) and physicochemical properties of honey (**A**) pH vs. honey area (%), (**B**) electrical conductivity (ms ∙ ppt) vs. honey area (%), (**C**) moisture content (% brix) vs. honey area (%), and (**D**) color (mm) vs. honey area (%).

Figure 8 illustrates relationships between honey area and the four physical parameters (pH, EC, moisture content, and color) to determine whether areas of honey impact these measurable physical properties. By analyzing the correlation, regarding to the Pearson correlation coefficient values, the honey areas estimated from all data-splitting schemes revealed the negative trends to the pH (r values range from −0.12 to −0.24) and moisture content (r values range from −0.16 to −0.17) whereas EC (r = 0.28–0.44) and color (r = 0.30–0.46) showed the positive trends. Especially, the 80:10:10 and 90:15:15 datasets were more positively or negatively correlated than the 70:15:15 dataset (Figure 8). The weak-moderate linear relationships between honey areas and physical parameters are shown in EC and color. These R^2^ results (R^2^ ≈ 0.2 for 80:10:10 and 90:15:15) provide insights into whether the honey area can serve as a predictive honey property for EC and color.

## 4. Discussion

### 4.1. Model Performance and Scalability

Higher resolutions (960 × 960, 800 × 800) with a 90:05:05 split yielded the best results, while lower resolutions reduced accuracy. Bounding box detection outperformed segmentation masks, especially for uncapped cells, though segmentation remained competitive at higher resolutions. Although the recommended 640 × 640 resolution was previously recommended and efficient, the accuracy analysis of capped honey areas is usually low because the capped honey areas are highly complex due to the layer of wax [13]. A larger and more diverse dataset would improve the generalization of the model, making it more robust in different conditions. In large-scale apiaries, optimized hardware and refined data acquisition strategies are essential for the practical application to manage the increased data volume effectively [35,36]. We also note that integrating cameras within hives requires more appropriate non-invasive methods to minimize disturbances to bee colonies because collecting data from a high number of beehives could increase disturbances during data capture, as the combs must be lifted and then replaced. However, this approach could yield a greater volume of input data for deep learning techniques to generate meaningful insights. While our method is suitable for small-scale farms, where the number of beehives ranges from 5 to 50 [4], we suggest that improvements in in-hive sensors and digital data processing from the hive environment are essential to minimize disturbance of the colony’s honeycombs. In addition, the computational resources are limited to the NVIDIA RTX 3070 laptop GPU, which limits the ability to train large models with higher-resolution images and more complex architectures.

The analysis of the honey area estimates across the different data splits provides a robust evaluation of the model’s consistency. As illustrated in Figure 7, the corelations from three distinct splits highlighted strong relationships as evidenced by the high correlation coefficients; r = 0.94 for 90:5:5 versus 80:10:10, r = 0.81 for 90:5:5 versus 70:15:15, and r = 0.81 for 80:10:10 versus 70:15:15. These comparisons also revealed statistically significant *p*-values, below the 0.05 threshold. The highest R^2^ with the value 0.84, *p* < 0.01 indicates that two data splits (90:5:5 versus 80:10:10) consistently captured the overall honey areas.

### 4.2. Association Between Regional Factors and Honey Quality Parameters

The results indicate that the honey area quantified via deep learning segmentation presents varied relationships with the physical parameters of honey. In Figure 8A, the pH of honey exhibits weak negative correlations with the honey area (ranged from −0.12 to −0.24) with low R^2^ values (0.05, 0.06 and 0.01, in 90:5:5, 80:10:10 and 70:15:15, respectively) and their *p*-values less than <0.05, indicating that the acid content is largely independent of the spatial extent of honey within the comb. This suggests that the acidity is predominantly determined by the intrinsic chemical composition of the nectar rather than by the extent of honey deposition in the comb. Previous studies have shown that floral origin and the organic acids present (e.g., gluconic acid) strongly influence honey pH [31,34,37,38]. Similarly, in Figure 8C, moisture content appears nearly unaffected by the honey area, with an R^2^ value below 0.03 and *p*-values exceeding 0.05. This result aligns with findings that moisture levels are mainly controlled by environmental conditions during nectar collection and post-harvest processing rather than by the spatial deposition of honey [31,33,37]. In contrast, in Figure 8B, electrical conductivity shows a modest relationship and gives positive correlation (R^2^ = 0.19, r ≈ 0.45, *p* < 0.05, except dataset 70:15:15), implying that larger honey areas may be associated with a slight increase in mineral and organic acid content. This observation is in line with external research indicating that darker honeys, which typically have higher mineral contents, also exhibit higher conductivity [32,33,39]. Furthermore, in Figure 8D, the color of honey demonstrates a moderate relationship with the honey area (R^2^ = 0.21, r ≈ 0.45, *p* < 0.05, except dataset 70:15:15). Honey color is widely recognized as an indicator of both floral source and mineral content [33,35]. Additional studies have confirmed that darker honeys generally contain higher levels of pigments and phenolic compounds, contributing to both color intensity and antioxidant capacity [39,40]. Overall, the method for detecting areas of honey is being improved via the automated image-based quantification, which offers a rapid and non-invasive tool for hive monitoring. However, the relationship between the honey area versus the physical parameters of honey is limited. The moderate correlations observed between electrical conductivity and color suggest that further refinement is needed. This aligns with previous findings indicating that honey characteristics are influenced by the nectar source [41,42]. As this study focuses on the physical properties of multifloral honeys, future work should aim to more precisely characterize honey samples. Subsequently, the current image-based protocol could be applied to both monofloral and multifloral honeys. This approach may provide valuable supplementary information and support the development of automated methods while maintaining high model accuracy. The weak correlations for pH and moisture content reinforce the necessity of combining image analysis with other direct physio-chemical measurements for a comprehensive evaluation of honey quality. Measuring sugar content may be one of the key nutrient factors [43] that could help in enabling a relationship to honey classifications via image processing.

Furthermore, combining the plant sources based on floral pollen identification and the nutrients in honey may help for impressive results to detect incoming honey quantity in honeybee colonies. Therefore, we recommend deploying such systems to progressively test during production phases, which does not measure the periods where the bees are hungry or dying due to dependence on physical properties of honey, thereby improving honeybee farm management and promoting sustainable apiculture practices.

## 5. Conclusions

The deep learning approach based on YOLOv11 has proven highly effective for the automated classification of honeycomb structures. When trained using high-resolution images (960 × 960 pixels) and an optimal dataset split of 90:5:5, the model achieved a mAP@0.5 of 83.4% for uncapped honey cells and 80.5% for capped honey cells. These results are due to image quality in enhancing classification accuracy. The linear regression analyses showed the weak relationships between the quantified honey area and the four physical parameters of honey, which can ultimately make it difficult to interpret the quality and efficiency of honey production. The moderate-weak parameters are conductivity and color, suggesting that these properties are primarily determined by color image-based measurements that provide supplementary insights into the honey’s color indicator.

## Figures and Tables

**Table 1:** Class features annotated in the image dataset.

Class	Description	Number of Annotation
Uncap	Polygon around the honey-uncapped cell	62,520
Cap	Polygon around the honey-capped cell	607
Other	Polygon around the area of the beeswax	300

**Table 2:** Preprocessing and augmentation were implemented on the dataset.

Category	Techniques Used	Description
Preprocessing	Auto-orient	Ensured all images were correctly oriented
	Resize	Stretched images to 960 × 960 pixels
	Auto-adjust contrast	Applied Adaptive Equalization for contrast
	Filter Null	Ensured all images contained annotations
Augmentation	Flip	Horizontal flipping
	Hue	Between −5° and +5°
	Saturation	Between −10% and +10%
	Brightness	Between −10% and +10%
	Exposure	Between −5% and +5%
	Noise	Up to 1% of the pixels

**Table 3:** Summary of mAP@0.5 from model training results based on image resolutions and data splitting.

Input Resolution	Dataset (Training: Validating: Testing)	mAP@0.5			
		Uncapped Honey Cells		Capped Honey Cells	
		Box	Mask	Box	Mask
960 × 960	90:5:5	0.834	0.743	0.805	0.805
	80:10:10	0.830	0.725	0.749	0.730
	70:15:15	0.777	0.634	0.659	0.635
800 × 800	90:5:5	0.842	0.513	0.775	0.681
	80:10:10	0.810	0.530	0.655	0.568
	70:15:15	0.774	0.482	0.604	0.618
640 × 640	90:5:5	0.773	0.434	0.790	0.720
	80:10:10	0.756	0.437	0.639	0.616
	70:15:15	0.709	0.405	0.591	0.547
512 × 512	90:5:5	0.655	0.273	0.685	0.638
	80:10:10	0.658	0.280	0.590	0.552
	70:15:15	0.597	0.255	0.559	0.582
256 × 256	90:5:5	0.229	0.028	0.505	0.378
	80:10:10	0.234	0.034	0.415	0.363
	70:15:15	0.209	0.028	0.428	0.466

## Data Availability

The data are available through a public repository on the GitHub platform, providing codes for the honeybee hive classification dataset. https://github.com/Panpakornk/honeybeehiveclassification (accessed on 12 January 2025); YOLOv11 available online at https://github.com/ultralytics/ultralytics (accessed on 12 January 2025).

## Supplementary Materials

The following supporting information can be downloaded at https://www.mdpi.com/article/10.3390/insects16060575/s1; Table S1. Physical parameter and image-processed parameters (honey area estimation from beehive); Table S2. Full image-processed parameters estimation in pixels (honey area estimation from the beehive).

## Abbreviations

The following abbreviations are used in this manuscript:

YOLO	You Only Look Once
AI	Artificial Intelligence
mAP	Mean average precision
mAP@0.5	Mean average precision calculated at an intersection over union (IoU) threshold of 0.50
RGB	Red, Green, Blue
R^2^	Coefficient of determination
r	Correlation coefficient
P	Statistical significance
