# Supplementary material for: Deep Learning-Based Detection of Honey Storage Areas in Apis mellifera Colonies for Predicting Physical Parameters of Honey via Linear Regression
Source: Insects. 2025 May 29;16(6):575. doi: 10.3390/insects16060575 (PMC12193066; doi:10.3390/insects16060575)
Supplement: Supplementary file 1 [file insects-16-00575-s001.zip › insects-3646717-supplementary.pdf]

Supplementary Materials

Deep Learning-Based Detection of Honey Storage Areas in *Apis mellifera* Colonies for Predicting Physical Parameters of Honey via Linear Regression

Watit Khokthong [1,2,3,†], Panpakorn Kritangkoon [3,†], Chainarong Sinpoo [1,2,4], Phuwasit Takioawong [2], Patcharin Phokasem [1,2,4] and Terd Disayathanoowat [1,2,*]

[1] Department of Biology, Faculty of Science, Chiang Mai University, Chiang Mai 50200, Thailand; watit.khokthong@cmu.ac.th (W.K.)
[2] Research Center of Deep Technology in Beekeeping and Bee Products for Sustainable Development Goals (SMART BEE SDGs), Chiang Mai University, Chiang Mai 50200, Thailand; chainarong.s@cmu.ac.th (C.S.); songkranoppa@gmail.com (P.T.)
[3] Bachelor of Environmental Science Program, Faculty of Science, Chiang Mai University, Chiang Mai 50200, Thailand, beckhaman@outlook.com (P.K.)
[4] Office of Research Administration, Chiang Mai University, Chiang Mai, 50200, Thailand; patcharin.ph@cmu.ac.th (P.P.)
† These authors contributed equally to this work.
* Correspondence: terd.dis@cmu.ac.th

Table S1. Physical parameter and image-processed parameters (honey area estimation from beehive).

Sample	Date	pH				Conductivity (mS · ppt)				Moisture Content (%Brix)				color (mm)				Honey area dataset 90:5:5 (%)	Honey area dataset 80:15:15 (%)	Honey area dataset 70:15:15 (%)
		1	2	3	Avg	1	2	3	Avg	1	2	3	Avg	1	2	3	Avg			
1	17-Jul-24	3.79	3.8	3.77	3.79	0.42	0.45	0.45	0.44	19.7	20.3	20.2	20.07	65	68	66	66.33	5.18	7.05	4.90
	18-Aug-24	3.8	3.79	3.8	3.80	0.36	0.36	0.36	0.36	22.4	22.4	22.4	22.40	48	48	47	47.67	10.73	12.25	11.41
2	17-Jul-24	4.31	4.32	4.31	4.31	0.68	0.67	0.67	0.67	19.1	18.9	18.9	18.97	93	93	92	92.67	4.97	4.43	4.18
	18-Aug-24	3.86	3.85	3.85	3.85	0.34	0.34	0.34	0.34	19	19	19	19.00	39	38	39	38.67	8.70	10.32	9.83
3	17-Jul-24	3.95	3.93	3.91	3.93	0.53	0.53	0.52	0.53	19.8	20.2	19.9	19.97	70	69	71	70.00	8.43	8.74	6.95
	18-Aug-24	3.92	3.93	3.87	3.91	0.35	0.35	0.35	0.35	18.7	18.7	18.7	18.70	43	43	42	42.67	9.90	9.13	9.34
4	17-Jul-24	3.85	3.85	3.84	3.85	0.47	0.47	0.47	0.47	20	20.2	20.2	20.13	63	66	66	65.00	8.42	10.71	9.63
	18-Aug-24	3.85	3.85	3.85	3.85	0.35	0.35	0.35	0.35	20.2	20.2	20.2	20.20	44	42	41	42.33	7.78	10.60	10.37

5	17-Jul-24	3.82	3.83	3.81	3.82	0.38	0.38	0.38	0.38	20.3	20.3	20.3	20.30	35	35	35	35.00	10.51	8.80	9.73
	18-Aug-24	3.83	3.82	3.83	3.83	0.32	0.32	0.32	0.32	22.2	22.2	22.2	22.20	36	37	35	36.00	7.56	6.33	7.07
6	17-Jul-24	3.78	3.79	3.79	3.79	0.4	0.39	0.38	0.39	19.6	19.7	19.7	19.67	32	32	32	32.00	7.74	7.85	4.83
	18-Aug-24	3.84	3.83	3.83	3.83	0.32	0.32	0.32	0.32	19	19	19	19.00	34	35	35	34.67	8.22	7.90	8.72
7	17-Jul-24	3.81	3.81	3.81	3.81	0.4	0.38	0.37	0.38	19.2	19.2	19.2	19.20	33	33	33	33.00	10.77	10.39	10.03
	18-Aug-24	3.81	3.86	3.85	3.84	0.32	0.32	0.32	0.32	19.4	19.4	19.4	19.40	40	41	40	40.33	10.23	10.60	12.01
8	17-Jul-24	3.82	3.81	3.81	3.81	0.38	0.38	0.38	0.38	18.9	19	18.9	18.93	32	31	31	31.33	2.34	2.49	0.53
	18-Aug-24	3.71	3.71	3.69	3.70	0.34	0.34	0.34	0.34	21.7	21.7	21.7	21.70	35	35	35	35.00	10.11	8.99	9.98
	18-Sep-24	3.83	3.83	3.83	3.83	0.22	0.22	0.22	0.22	15.8	15.8	15.8	15.80	3	3	3	3.00	12.10	11.67	11.64
9	20-Jul-24	3.85	3.85	3.85	3.85	0.51	0.51	0.51	0.51	19.9	19.9	19	19.60	69	69	69	69.00	21.88	15.11	14.64
	18-Aug-24	3.76	3.77	3.77	3.77	0.39	0.39	0.39	0.39	21.3	21.3	21.3	21.30	43	43	43	43.00	13.29	13.94	13.72
	18-Sep-24	3.52	3.52	3.52	3.52	0.27	0.27	0.27	0.27	30.4	30.4	30.4	30.40	21	21	21	21.00	7.22	7.89	4.18
	26-Nov-24	3.73	3.73	3.73	3.73	0.3	0.3	0.3	0.30	17.5	17.5	17.5	17.50	23	23	23	23.00	14.15	14.01	13.80
10	17-Jul-24	3.75	3.75	3.74	3.75	0.39	0.39	0.39	0.39	20.8	20.8	20.8	20.80	45	45	45	45.00	15.92	17.01	10.44
	18-Aug-24	3.76	3.75	3.76	3.76	0.37	0.37	0.37	0.37	20	20	20	20.00	43	45	42	43.33	11.10	12.29	7.39
	18-Sep-24	3.54	3.54	3.54	3.54	0.26	0.26	0.26	0.26	30.5	30.5	30.5	30.50	25	25	25	25.00	3.95	4.52	5.57
	26-Nov-24	3.7	3.7	3.7	3.70	0.3	0.3	0.3	0.30	17.4	17.4	17.4	17.40	15	15	15	15.00	24.78	25.23	24.38
11	17-Jul-24	3.8	3.8	3.8	3.80	0.41	0.41	0.41	0.41	20.1	20.1	20.1	20.10	53	53	53	53.00	11.36	10.57	9.89
	18-Aug-24	3.77	3.77	3.78	3.77	0.4	0.4	0.4	0.40	19.1	19.1	19.1	19.10	52	52	51	51.67	10.51	11.05	12.42
	18-Sep-24	3.51	3.51	3.51	3.51	0.27	0.27	0.27	0.27	30	30	30	30.00	20	20	20	20.00	1.30	1.48	0.73
	26-Nov-24	3.72	3.72	3.72	3.72	0.31	0.31	0.31	0.31	19	19	19	19.00	24	24	24	24.00	6.62	5.29	7.48
12	17-Jul-24	3.64	3.69	3.62	3.65	0.36	0.36	0.36	0.36	20.2	20.2	20.2	20.20	37	37	37	37.00	11.60	15.84	11.09
	18-Aug-24	3.72	3.71	3.71	3.71	0.36	0.36	0.36	0.36	20.7	20.7	20.7	20.70	47	47	48	47.33	7.28	18.47	9.97
	18-Sep-24	3.5	3.5	3.5	3.50	0.28	0.28	0.28	0.28	30.5	30.5	30.5	30.50	24	24	24	24.00	6.04	5.36	5.51
13	17-Jul-24	3.67	3.72	3.65	3.68	0.4	0.4	0.4	0.40	21.2	21.3	21.3	21.27	59	59	58	58.67	14.16	13.26	12.64
	18-Aug-24	3.81	3.81	3.79	3.80	0.49	0.49	0.49	0.49	21.6	21.6	21.6	21.60	91	91	91	91.00	12.41	12.51	12.85
	18-Sep-24	3.54	3.54	3.54	3.54	0.38	0.38	0.38	0.38	25	25	25	25.00	86	86	86	86.00	14.74	15.13	15.60
14	17-Jul-24	3.61	3.66	3.59	3.62	0.39	0.39	0.39	0.39	20.2	20.3	20.3	20.27	62	61	61	61.33	19.00	18.15	13.99
	18-Aug-24	3.64	3.63	3.63	3.63	0.41	0.41	0.41	0.41	19	19	19	19.00	72	71	71	71.33	19.13	18.58	20.33
	18-Sep-24	3.44	3.44	3.44	3.44	0.35	0.35	0.35	0.35	25.3	25.3	25.3	25.30	68	68	68	68.00	17.28	15.99	15.60

15	17-Jul-24	3.75	3.8	3.73	3.76	0.51	0.51	0.51	0.51	20.7	20.7	20.7	20.70	79	80	78	79.00	9.98	10.12	7.51
	18-Aug-24	3.69	3.66	3.67	3.67	0.4	0.4	0.4	0.40	18.2	18.2	18.2	18.20	77	78	75	76.67	20.64	16.45	17.54
	18-Sep-24	3.46	3.46	3.46	3.46	0.31	0.31	0.31	0.31	30.4	30.4	30.4	30.40	37	37	37	37.00	12.52	12.60	12.49
	26-Nov-24	3.69	3.69	3.69	3.69	0.3	0.3	0.3	0.30	18.6	18.6	18.6	18.60	23	23	23	23.00	20.67	18.98	18.80
16	17-Jul-24	3.67	3.72	3.65	3.68	0.44	0.44	0.44	0.44	19.3	19.7	19.9	19.63	70	70	70	70.00	15.44	14.94	15.81
	18-Aug-24	3.62	3.6	3.59	3.60	0.41	0.41	0.41	0.41	22.2	22.2	22.2	22.20	77	77	76	76.67	15.36	16.17	15.56
	18-Sep-24	3.48	3.48	3.48	3.48	0.33	0.33	0.33	0.33	26.1	26.1	26.1	26.10	45	45	45	45.00	12.44	11.32	7.66
17	19-Jul-24	4.25	4.23	4.26	4.25	0.19	0.19	0.19	0.19	19.4	19.4	19.4	19.40	7	7	8	7.33	7.99	8.84	5.91
	19-Aug-24	4.14	4.13	4.13	4.13	0.17	0.17	0.17	0.17	19.3	19.3	19.3	19.30	5	5	5	5.00	5.37	7.06	6.75
	18-Sep-24	3.56	3.56	3.56	3.56	0.21	0.21	0.21	0.21	21.1	21.1	21.1	21.10	15	15	15	15.00	2.61	3.01	2.00
18	19-Jul-24	4.4	4.41	4.4	4.40	0.18	0.18	0.18	0.18	19	19	19	19.00	5	5	5	5.00	6.41	6.67	4.32
	19-Aug-24	4.26	4.22	4.22	4.23	0.16	0.16	0.16	0.16	18.5	18.5	18.5	18.50	6	6	6	6.00	5.84	7.74	4.20
	18-Sep-24	3.55	3.55	3.55	3.55	0.22	0.22	0.22	0.22	22.6	22.6	22.6	22.60	18	18	18	18.00	3.63	3.81	3.56
19	19-Jul-24	4.25	4.27	4.28	4.27	0.17	0.17	0.17	0.17	18.5	18.6	18.6	18.57	7	7	7	7.00	11.91	10.88	11.68
	19-Aug-24	4.26	4.22	4.24	4.24	0.16	0.16	0.16	0.16	18.7	18.7	18.7	18.70	7	7	7	7.00	10.78	8.38	10.18
	18-Sep-24	3.55	3.55	3.55	3.55	0.22	0.22	0.22	0.22	23.4	23.4	23.4	23.40	19	19	19	19.00	8.60	8.90	9.80
20	19-Jul-24	4.27	4.29	4.3	4.29	0.17	0.17	0.17	0.17	19	19	19	19.00	7	7	7	7.00	11.34	11.96	38.61
	19-Aug-24	4.19	4.19	4.19	4.19	0.16	0.16	0.16	0.16	27.9	27.9	27.9	27.90	4	4	4	4.00	13.20	11.76	13.33
	18-Sep-24	3.56	3.56	3.56	3.56	0.21	0.21	0.21	0.21	21.8	21.8	21.8	21.80	18	18	18	18.00	5.70	5.11	5.76
21	19-Jul-24	4.06	4.08	4.09	4.08	0.21	0.21	0.21	0.21	20	19.6	19.4	19.67	26	32	26	28.00	4.66	4.90	5.33
	19-Aug-24	4.09	4.07	4.07	4.08	0.18	0.18	0.18	0.18	27.6	27.6	27.6	27.60	14	13	14	13.67	2.22	2.15	1.88
	18-Sep-24	3.59	3.59	3.59	3.59	0.21	0.21	0.21	0.21	20.6	20.6	20.6	20.60	23	23	23	23.00	0.46	0.61	0.36
22	19-Jul-24	4.12	4.14	4.15	4.14	0.21	0.21	0.21	0.21	18.6	18.5	18.5	18.53	32	29	29	30.00	4.32	4.99	2.83
	19-Aug-24	4.09	4.12	4.1	4.10	0.18	0.18	0.18	0.18	20.9	20.9	20.9	20.90	11	11	12	11.33	5.44	5.41	5.72
	18-Sep-24	3.6	3.6	3.6	3.60	0.22	0.22	0.22	0.22	19.8	19.8	19.8	19.80	20	20	20	20.00	1.02	1.00	0.50
23	19-Jul-24	4.22	4.24	4.25	4.24	0.19	0.19	0.19	0.19	18.1	18.2	18.2	18.17	21	21	21	21.00	3.96	4.22	0.73
	19-Aug-24	4.04	4.03	4.04	4.04	0.18	0.18	0.18	0.18	28.1	28.1	28.1	28.10	15	15	15	15.00	2.40	2.11	1.51
	18-Sep-24	3.61	3.61	3.61	3.61	0.21	0.21	0.21	0.21	21.4	21.4	21.4	21.40	22	22	22	22.00	1.11	0.95	0.71
24	19-Jul-24	4.2	4.22	4.23	4.22	0.21	0.21	0.21	0.21	19.7	19.8	19.8	19.77	46	45	45	45.33	9.14	12.48	9.52
	19-Aug-24	4.09	4.08	4.08	4.08	0.18	0.18	0.18	0.18	19.4	19.4	19.4	19.40	20	20	20	20.00	8.92	9.35	9.42

	18-Sep-24	3.62	3.62	3.62	3.62	0.21	0.21	0.21	0.21	20.4	20.4	20.4	20.40	20	20	20	20.00	1.84	2.17	1.16
25	19-Jul-24	4.36	4.37	4.38	4.37	0.17	0.17	0.17	0.17	19.1	19.2	19.2	19.17	6	8	9	7.67	6.33	4.57	4.54
	19-Aug-24	4.17	4.17	4.16	4.17	0.18	0.18	0.18	0.18	20.3	20.3	20.3	20.30	3	3	3	3.00	3.81	3.84	4.71
26	19-Jul-24	4.33	4.34	4.34	4.34	0.18	0.18	0.18	0.18	19.1	19.2	19.2	19.17	8	9	8	8.33	8.41	8.29	6.23
	19-Aug-24	4.24	4.21	4.21	4.22	0.18	0.18	0.18	0.18	19.6	19.6	19.6	19.60	5	5	5	5.00	2.61	3.13	2.68
27	19-Jul-24	4.23	4.24	4.25	4.24	0.19	0.19	0.19	0.19	19	19	19.1	19.03	13	12	14	13.00	13.27	12.88	10.27
	19-Aug-24	4.14	4.13	4.13	4.13	0.19	0.19	0.19	0.19	20.3	20.3	20.3	20.30	9	9	9	9.00	9.78	9.08	11.23
28	19-Jul-24	4.36	4.37	4.38	4.37	0.16	0.16	0.16	0.16	18.9	18.9	18.9	18.90	14	16	12	14.00	5.45	5.46	4.28
29	19-Aug-24	3.94	3.95	3.95	3.95	0.19	0.19	0.19	0.19	21.1	21.1	21.1	21.10	10	11	9	10.00	3.03	3.59	0.63
	26-Nov-24	3.81	3.81	3.81	3.81	0.18	0.18	0.18	0.18	19.3	19.3	19.3	19.30	2	2	2	2.00	4.29	6.35	5.70
30	19-Jul-24	4.28	4.29	4.3	4.29	0.18	0.18	0.18	0.18	20.7	20.7	20.7	20.70	7	6	6	6.33	1.17	1.32	1.19
	19-Aug-24	4.11	4.11	4.11	4.11	0.18	0.18	0.18	0.18	20	20	20	20.00	11	11	11	11.00	10.78	10.24	10.36
31	19-Jul-24	4.27	4.28	4.29	4.28	0.18	0.18	0.18	0.18	21	21	21	21.00	5	5	5	5.00	7.93	8.52	7.36
	19-Aug-24	4.09	4.09	4.08	4.09	0.18	0.18	0.18	0.18	20.3	20.3	20.3	20.30	13	13	13	13.00	6.02	6.17	5.20
32	19-Aug-24	4.03	4.03	4.03	4.03	0.19	0.19	0.19	0.19	20.4	20.4	20.4	20.40	18	18	18	18.00	3.19	3.88	3.85

Table S2. Full image-processed parameters estimation in pixel (honey area estimation from beehive)

Sample	Date	Train 90/5/5					Train 80/10/10					Train 70/15/15				
		Area (Pixel)				%	Area (Pixel)				%	Area (Pixel)				%
		A	B	SUM	total		A	B	SUM	total		A	B	SUM	total	
1	17-Jul-24	16224	12687	28911	278938	5.18	21197	18528	39725	281538	7.05	15854	11773	27627	281665	4.90
	18-Aug-24	29690	30172	59862	278938	10.73	30249	30735	60984	248816	12.25	30083	27869	57952	253968	11.41
	20-Sep-24	2288	4874	7162	278938	1.28	3526	7720	11246	271236	2.07	3472	7046	10518	240187	2.19
	26-Nov-24	30959	31637	62596	278938	11.22	42054	41606	83660	346718	12.06	42087	40912	82999	353983	11.72
2	17-Jul-24	20447	7273	27720	278938	4.97	18368	7217	25585	288804	4.43	16600	7590	24190	289094	4.18
	18-Aug-24	29874	18668	48542	278938	8.70	30887	21512	52399	253832	10.32	31281	19584	50865	258658	9.83
	20-Sep-24	2687	16403	19090	278938	3.42	6562	21372	27934	271236	5.15	6650	19116	25766	269728	4.78
	26-Nov-24	10090	3595	13685	278938	2.45	14471	4786	19257	346718	2.78	16094	5104	21198	333586	3.18
3	17-Jul-24	27935	19116	47051	278938	8.43	28438	23434	51872	296712	8.74	28076	13501	41577	298914	6.95
	18-Aug-24	35214	20041	55255	278938	9.90	33758	21550	55308	303012	9.13	36660	19556	56216	301003	9.34
	20-Sep-24	21718	2780	24498	278938	4.39	22972	7469	30441	287864	5.29	28713	2808	31521	289847	5.44
	26-Nov-24	6290	12835	19125	278938	3.43	8977	17097	26074	337594	3.86	8535	15525	24060	336299	3.58
4	17-Jul-24	23198	23749	46947	278938	8.42	37768	23339	61107	285412	10.71	32361	22781	55142	286191	9.63

	18-Aug-24	17901	25481	43382	278938	7.78	22098	33293	55391	261342	10.60	21453	33523	54976	265067	10.37
	20-Sep-24	17481	10455	27936	278938	5.01	22244	13096	35340	292446	6.04	20520	13398	33918	293832	5.77
	26-Nov-24	13369	31030	44399	278938	7.96	3301	18028	21329	323284	3.30	9647	25464	35111	325412	5.39
5	17-Jul-24	24513	34133	58646	278938	10.51	17361	33905	51266	291151	8.80	25540	32023	57563	295758	9.73
	18-Aug-24	9776	32423	42199	278938	7.56	8631	27269	35900	283782	6.33	7007	33033	40040	283060	7.07
	20-Sep-24	19619	16997	36616	278938	6.56	22116	18657	40773	276958	7.36	21716	11240	32956	279649	5.89
	26-Nov-24	38378	8777	47155	278938	8.45	39632	10196	49828	326079	7.64	38205	9732	47937	317420	7.55
6	17-Jul-24	21292	21862	43154	278938	7.74	17428	28931	46359	295133	7.85	8680	19473	28153	291400	4.83
	18-Aug-24	23791	22071	45862	278938	8.22	26231	16831	43062	272591	7.90	25658	22311	47969	275157	8.72
	20-Sep-24	1444	3609	5053	278938	0.91	1921	2245	4166	271605	0.77	424	1828	2252	270608	0.42
	26-Nov-24	39082	23422	62504	278938	11.20	39973	21772	61745	337348	9.15	38759	25044	63803	336795	9.47
7	17-Jul-24	34827	31169	65996	306302	10.77	29176	34777	63953	307665	10.39	26600	34525	61125	304702	10.03
	18-Aug-24	27638	28471	56109	274121	10.23	27169	30952	58121	274064	10.60	36471	30448	66919	278484	12.01
	20-Sep-24	21012	16746	37758	260439	7.25	18737	14827	33564	259921	6.46	16077	11529	27606	260897	5.29
	26-Nov-24	34705	35032	69737	332471	10.49	34491	35349	69840	331404	10.54	37467	41194	78661	333250	11.80
8	17-Jul-24	7160	6123	13283	283693	2.34	7212	6877	14089	283020	2.49	133	2853	2986	282684	0.53
	18-Aug-24	22495	27904	50399	249329	10.11	19446	28386	47832	266126	8.99	25156	29243	54399	272550	9.98

	20-Sep-24	N/A	N/A	N/A	N/A	N/A	N/A	N/A	N/A	N/A	N/A	N/A	N/A	N/A	N/A	N/A
	26-Nov-24	38340	38658	76998	318270	12.10	35829	39108	74937	320992	11.67	38377	36932	75309	323595	11.64
9	17-Jul-24	43611	80391	124002	283395	21.88	41775	43814	85589	283267	15.11	41187	41190	82377	281333	14.64
	18-Aug-24	24733	53565	78298	294564	13.29	26889	55016	81905	293715	13.94	25805	53781	79586	289984	13.72
	20-Sep-24	14743	17205	31948	221220	7.22	17689	17567	35256	223552	7.89	1613	17163	18776	224432	4.18
	26-Nov-24	51082	40079	91161	322025	14.15	50736	39875	90611	323283	14.01	48726	40863	89589	324481	13.80
10	17-Jul-24	43663	47126	90789	285082	15.92	43441	53176	96617	283953	17.01	40801	55597	96398	461581	10.44
	18-Aug-24	30797	34230	65027	292984	11.10	31250	40896	72146	293451	12.29	28888	39537	68425	462670	7.39
	20-Sep-24	11276	9100	20376	257656	3.95	12977	11679	24656	272603	4.52	13234	17163	30397	273070	5.57
	26-Nov-24	83080	77450	160530	323943	24.78	86593	77912	164505	326075	25.23	88273	76153	164426	337224	24.38
11	17-Jul-24	32759	34997	67756	298344	11.36	28422	34318	62740	296897	10.57	31644	37020	68664	347278	9.89
	18-Aug-24	32620	27590	60210	286572	10.51	32879	31646	64525	291876	11.05	31982	27245	59227	238473	12.42
	20-Sep-24	1268	4729	5997	229904	1.30	533	6814	7347	247792	1.48	381	3935	4316	294682	0.73
	26-Nov-24	29079	16814	45893	346377	6.62	27341	9140	36481	344854	5.29	27378	17331	44709	298791	7.48
12	17-Jul-24	43875	22560	66435	286378	11.60	75820	27609	103429	326571	15.84	35238	27988	63226	285131	11.09
	18-Aug-24	7875	28327	36202	248719	7.28	94245	29844	124089	335983	18.47	27176	26906	54082	271209	9.97
	20-Sep-24	17586	9329	26915	222963	6.04	14920	9227	24147	225289	5.36	15755	9319	25074	227699	5.51

	26-Nov-24	61396	12051	73447	324809	11.31	62907	168	63075	320110	9.85	60899	11321	72220	325354	11.10
13	17-Jul-24	54526	29471	83997	296667	14.16	53830	25660	79490	299816	13.26	50685	24299	74984	296726	12.64
	18-Aug-24	47887	18337	66224	266893	12.41	49365	18534	67899	271481	12.51	48343	20531	68874	267985	12.85
	20-Sep-24	41646	40202	81848	277667	14.74	44095	41919	86014	284279	15.13	45359	42442	87801	281415	15.60
	26-Nov-24	8482	19245	27727	314804	4.40	9473	15086	24559	318973	3.85	9660	21708	31368	317660	4.94
14	17-Jul-24	58218	45298	103516	272437	19.00	55772	45832	101604	279960	18.15	52795	24299	77094	275479	13.99
	18-Aug-24	44041	47969	92010	240494	19.13	47133	43849	90982	244880	18.58	49046	50408	99454	244657	20.33
	20-Sep-24	40459	52787	93246	269795	17.28	37513	49551	87064	272298	15.99	35418	50129	85547	274259	15.60
	26-Nov-24	15786	13380	29166	329111	4.43	16601	17125	33726	333180	5.06	22055	20718	42773	335248	6.38
15	17-Jul-24	33728	22132	55860	279736	9.98	31064	27034	58098	287096	10.12	30333	12647	42980	286119	7.51
	18-Aug-24	76508	44346	120854	292808	20.64	38353	45703	84056	255472	16.45	40232	49694	89926	256306	17.54
	20-Sep-24	33872	36617	70489	281407	12.52	36176	32561	68737	272771	12.60	31455	37627	69082	276632	12.49
	26-Nov-24	45639	79697	125336	303246	20.67	33535	80218	113753	299696	18.98	33452	78357	111809	297356	18.80
16	17-Jul-24	55358	32356	87714	284053	15.44	54203	31048	85251	285312	14.94	54470	35611	90081	284837	15.81
	18-Aug-24	38587	38754	77341	251720	15.36	37738	44712	82450	255015	16.17	37394	40769	78163	251123	15.56
	20-Sep-24	33648	33212	66860	268686	12.44	34202	26918	61120	269984	11.32	29443	27224	56667	369819	7.66
	26-Nov-24	42886	39755	82641	325474	12.70	43504	22868	66372	326571	10.16	43604	29127	72731	325229	11.18

17	19-Jul-24	20256	25010	45266	283137	7.99	19420	30881	50301	284413	8.84	10612	22307	32919	278598	5.91
	19-Aug-24	13893	13233	27126	252716	5.37	22468	13500	35968	254644	7.06	21351	13015	34366	254442	6.75
	20-Sep-24	5701	7508	13209	253038	2.61	6931	8352	15283	254157	3.01	5663	4569	10232	255821	2.00
	26-Nov-24	147	0	147	288142	0.03	0	0	0	289797	0.00	0	0	0	289567	0.00
18	19-Jul-24	6419	27576	33995	265356	6.41	3857	33204	37061	277727	6.67	5204	18782	23986	277656	4.32
	19-Aug-24	8617	21029	29646	253919	5.84	22468	16967	39435	254644	7.74	7252	14066	21318	254050	4.20
	20-Sep-24	6380	11632	18012	248328	3.63	6931	12458	19389	254157	3.81	4177	12959	17136	240703	3.56
	26-Nov-24	0	0	0	287420	0.00	0	158	158	290323	0.03	72	0	72	287393	0.01
19	19-Jul-24	35579	32251	67830	284732	11.91	29981	30930	60911	280048	10.88	34938	31747	66685	285545	11.68
	19-Aug-24	27174	32521	59695	276761	10.78	26560	16980	43540	259876	8.38	29149	24402	53551	262931	10.18
	20-Sep-24	18359	24600	42959	249879	8.60	21352	24495	45847	257600	8.90	24435	26599	51034	260473	9.80
	26-Nov-24	0	0	0	292717	0.00	0	0	0	289622	0.00	389	434	823	290173	0.14
20	19-Jul-24	32963	32466	65429	288375	11.34	33752	32653	66405	277584	11.96	35912	33723	69635	90185	38.61
	19-Aug-24	29601	43644	73245	277495	13.20	21665	44106	65771	279635	11.76	31653	44566	76219	285879	13.33
	20-Sep-24	13605	16150	29755	260833	5.70	13718	13009	26727	261477	5.11	14157	16068	30225	262561	5.76
	26-Nov-24	1463	0	1463	294818	0.25	2024	0	2024	298629	0.34	2637	152	2789	297423	0.47
21	19-Jul-24	16929	12055	28984	310919	4.66	15865	14944	30809	314318	4.90	19017	14146	33163	311213	5.33

	19-Aug-24	5068	7436	12504	282169	2.22	3958	8857	12815	298625	2.15	3500	7664	11164	297171	1.88
	20-Sep-24	2421	144	2565	278055	0.46	2225	1249	3474	284160	0.61	1450	607	2057	281799	0.36
	26-Nov-24	2043	941	2984	332918	0.45	1109	1235	2344	338710	0.35	514	244	758	335661	0.11
22	19-Jul-24	10429	12303	22732	262845	4.32	9104	19729	28833	288804	4.99	6418	8549	14967	264296	2.83
	19-Aug-24	11383	17994	29377	270051	5.44	11113	16328	27441	253832	5.41	10394	20470	30864	269762	5.72
	20-Sep-24	2381	2789	5170	253076	1.02	2099	2954	5053	252815	1.00	911	1582	2493	251282	0.50
	26-Nov-24	0	0	0	291754	0.00	154	0	154	298013	0.03	317	0	317	298167	0.05
23	19-Jul-24	17995	5942	23937	302118	3.96	20955	4674	25629	303948	4.22	3249	1135	4384	300208	0.73
	19-Aug-24	9014	4577	13591	283584	2.40	8799	3291	12090	286059	2.11	6318	2258	8576	284636	1.51
	20-Sep-24	2891	3011	5902	266319	1.11	2684	2409	5093	268945	0.95	2309	1482	3791	267999	0.71
	26-Nov-24	0	0	0	344102	0.00	0	0	0	350857	0.00	3999	167	4166	349141	0.60
24	19-Jul-24	25780	21244	47024	257196	9.14	35885	30134	66019	264402	12.48	28149	20863	49012	257373	9.52
	19-Aug-24	29252	17725	46977	263181	8.92	31039	19965	51004	272663	9.35	31942	21589	53531	284194	9.42
	20-Sep-24	6605	2641	9246	251557	1.84	6151	4816	10967	252700	2.17	4926	912	5838	252727	1.16
	26-Nov-24	2138	2172	4310	286891	0.75	4154	4718	8872	287573	1.54	3931	3468	7399	286553	1.29
25	19-Jul-24	8622	24651	33273	262878	6.33	8678	15569	24247	265084	4.57	7595	16438	24033	264925	4.54
	19-Aug-24	7603	13168	20771	272556	3.81	10167	11012	21179	275657	3.84	9254	16690	25944	275150	4.71

	20-Sep-24	651	11648	12299	255583	2.41	262	11689	11951	255685	2.34	953	9584	10537	256500	2.05
	26-Nov-24	35146	36596	71742	309623	11.59	28618	31048	59666	308331	9.68	35852	32708	68560	312808	10.96
26	19-Jul-24	22344	24161	46505	276513	8.41	22996	22691	45687	275483	8.29	16437	18374	34811	279457	6.23
	19-Aug-24	11627	3328	14955	286460	2.61	14743	3232	17975	286952	3.13	12049	3445	15494	288747	2.68
	20-Sep-24	763	216	979	263987	0.19	1366	386	1752	268648	0.33	1640	397	2037	268012	0.38
	26-Nov-24	192	0	192	352394	0.03	350	0	350	355781	0.05	192	168	360	353374	0.05
27	19-Jul-24	35189	35794	70983	267547	13.27	36039	33303	69342	269147	12.88	25680	30620	56300	274212	10.27
	19-Aug-24	22513	30942	53455	273163	9.78	23137	27071	50208	276498	9.08	29182	34177	63359	282016	11.23
	20-Sep-24	5292	10754	16046	257055	3.12	8421	10613	19034	255663	3.72	12546	16307	28853	259639	5.56
	26-Nov-24	39088	38938	78026	303949	12.84	40250	39029	79279	313196	12.66	39961	39357	79318	307015	12.92
28	19-Jul-24	21155	6695	27850	255313	5.45	20920	7288	28208	258269	5.46	17648	4651	22299	260588	4.28
	19-Aug-24	1569	0	1569	248757	0.32	2213	0	2213	265855	0.42	2198	415	2613	257386	0.51
	20-Sep-24	299	299	598	237385	0.13	441	346	787	240993	0.16	151	0	151	241083	0.03
	26-Nov-24	31157	34445	65602	303057	10.82	14383	23885	38268	298379	6.41	25220	36160	61380	477585	6.43
29	19-Jul-24	360	1492	1852	256739	0.36	4771	3858	8629	260353	1.66	1267	1962	3229	257562	0.63
	19-Aug-24	0	16214	16214	267520	3.03	245	19161	19406	269966	3.59	0	14651	14651	268928	2.72
	20-Sep-24	99	0	99	242082	0.02	857	0	857	244052	0.18	105	0	105	243033	0.02

	26-Nov-24	9890	15234	25124	292912	4.29	12572	24604	37176	292945	6.35	11888	21630	33518	294140	5.70
30	19-Jul-24	2590	3328	5918	253546	1.17	2570	4111	6681	252328	1.32	3126	2951	6077	254777	1.19
	19-Aug-24	23707	34078	57785	267979	10.78	19994	35375	55369	270276	10.24	21370	35312	56682	273491	10.36
	20-Sep-24	2723	3509	6232	235220	1.32	2760	3849	6609	235794	1.40	2941	3293	6234	237513	1.31
	26-Nov-24	33645	23432	57077	288316	9.90	38249	27735	65984	292969	11.26	34832	24948	59780	294165	10.16
31	19-Jul-24	22544	18539	41083	259107	7.93	23187	21020	44207	259499	8.52	23293	15545	38838	263707	7.36
	19-Aug-24	19877	14018	33895	281340	6.02	21137	13931	35068	284097	6.17	19439	10044	29483	283421	5.20
	20-Sep-24	1982	130	2112	240187	0.44	2626	0	2626	242698	0.54	2228	0	2228	241876	0.46
	26-Nov-24	24831	28808	53639	309417	8.67	25191	28283	53474	311479	8.58	26985	33609	60594	301150	10.06
32	19-Jul-24	2980	1418	4398	262639	0.84	8675	7820	16495	264995	3.11	3848	4219	8067	263017	1.53
	19-Aug-24	5220	12769	17989	282256	3.19	8514	13514	22028	284061	3.88	5400	16195	21595	280812	3.85
	20-Sep-24	227	0	227	242848	0.05	434	0	434	245325	0.09	233	604	837	243379	0.17
	26-Nov-24	2868	3403	6271	323376	0.97	2783	7073	9856	323773	1.52	5339	17193	22532	550737	2.05
